# Metabolic versatility in *Haemophilus influenzae*: a metabolomic and genomic analysis

**DOI:** 10.3389/fmicb.2014.00069

**Published:** 2014-03-04

**Authors:** Dk Seti Maimonah Pg Othman, Horst Schirra, Alastair G. McEwan, Ulrike Kappler

**Affiliations:** ^1^School of Chemistry and Molecular Biosciences, The University of QueenslandSt. Lucia, QLD, Australia; ^2^Centre for Advanced Imaging, The University of QueenslandSt. Lucia, QLD, Australia

**Keywords:** *Haemophilus influenzae*, carbon metabolism, proton NMR, gene expression, enzymes

## Abstract

*Haemophilus influenzae* is a host adapted human pathogen known to contribute to a variety of acute and chronic diseases of the upper and lower respiratory tract as well as the middle ear. At the sites of infection as well as during growth as a commensal the environmental conditions encountered by *H. influenzae* will vary significantly, especially in terms of oxygen availability, however, the mechanisms by which the bacteria can adapt their metabolism to cope with such changes have not been studied in detail. Using targeted metabolomics the spectrum of metabolites produced during growth of *H. influenzae* on glucose in RPMI-based medium was found to change from acetate as the main product during aerobic growth to formate as the major product during anaerobic growth. This change in end-product is likely caused by a switch in the major route of pyruvate degradation. Neither lactate nor succinate or fumarate were major products of *H. influenzae* growth under any condition studied. Gene expression studies and enzyme activity data revealed that despite an identical genetic makeup and very similar metabolite production profiles, *H. influenzae* strain Rd appeared to favor glucose degradation via the pentose phosphate pathway, while strain 2019, a clinical isolate, showed higher expression of enzymes involved in glycolysis. Components of the respiratory chain were most highly expressed during microaerophilic and anaerobic growth in both strains, but again clear differences existed in the expression of genes associated e.g., with NADH oxidation, nitrate and nitrite reduction in the two strains studied. Together our results indicate that *H. influenzae* uses a specialized type of metabolism that could be termed “respiration assisted fermentation” where the respiratory chain likely serves to alleviate redox imbalances caused by incomplete glucose oxidation, and at the same time provides a means of converting a variety of compounds including nitrite and nitrate that arise as part of the host defence mechanisms.

## Introduction

*Haemophilus influenzae* is a host-adapted human pathogen that occurs naturally in the nasopharynx of healthy children and adults (Kuklinska and Kilian, 1984). However, it is also involved in many acute and chronic infections such as otitis media, asthma and chronic obstructive pulmonary disease (COPD) (Karasic et al., 1989; Berkovitch et al., 2002; Revai et al., 2008; Essilfie et al., 2011, 2012; Moghaddam et al., 2011). The occurrence of these infections is linked to the ability of *H. influenzae* to persist in different niches in the human body, including the lung, nasopharynx, and the middle ear. These niches vary in carbon metabolite and oxygen availability and also in ambient pH. For example, while the lung is a mostly aerobic environment (Worlitzsch et al., 2002) the middle ear is almost completely anaerobic (Luntz et al., 1995; Sade et al., 1995). It follows that *H. influenzae*, like other bacterial pathogens, should possess a repertoire of metabolic pathways that enables it to survive in the variety of the environments that it encounters in the human host.

Recent studies on pathogens such as *Mycobacterium* species (Rhee et al., 2011), *Legionella pneumophila* (Edwards et al., 2009, 2013), and *Escherichia coli* (Alteri and Mobley, 2012) have demonstrated clearly that subtle changes in pathway composition or enzyme activities affect virulence of these bacteria. For example, *Mycobacterium bovis* showed attenuation in mice when it lacked phosphenolpyruvate (PEP) carboxykinase, an enzyme involved in the synthesis of PEP from oxaloacetate (Liu et al., 2003). Similarly, the glyoxylate cycle and the TCA cycle enzymes isocitrate lyase and succinate dehydrogenase have been shown to be critical for virulence and persistence of *Mycobacterium tuberculosis* and *E. coli* (McKinney et al., 2000; Alteri and Mobley, 2012). The metabolic processes underpinning the ability of *H. influenzae* to persist in the human body have never been studied in depth and analysis of metabolism in this bacterium has been limited mostly to genome reconstructions and the generation of *in silico* models of metabolic fluxes (Edwards and Palsson, 1999; Schilling and Palsson, 2000; Schilling et al., 2000; Papin et al., 2002; Raghunathan et al., 2004).

**Figure 1:**
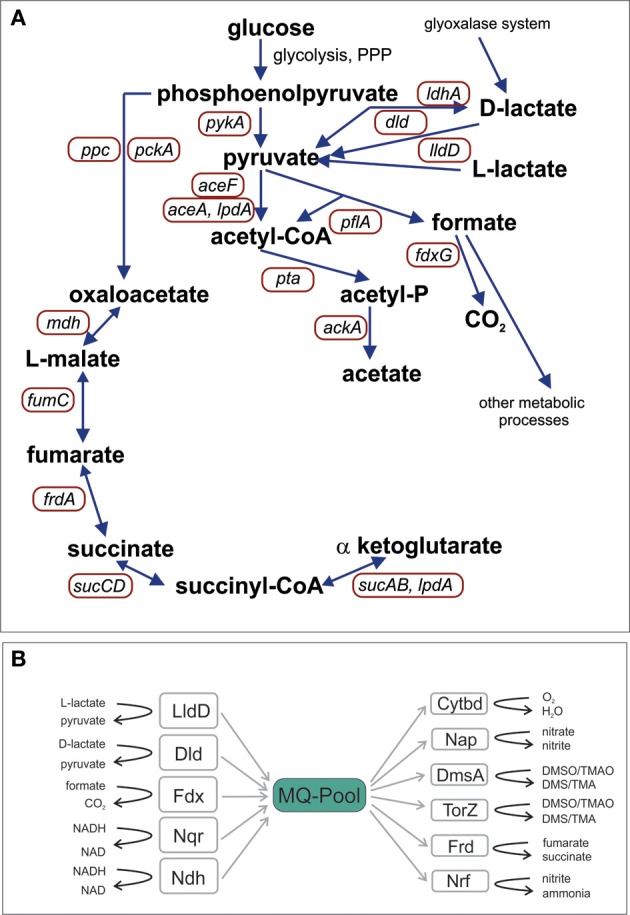
**Schematic representation of *H. influenzae* pathways for central carbon metabolism (A) and the elements of the respiratory chain (B).** Abbreviations: pyruvate dehydrogenase complex, *aceF*; Pyruvate formate lyase, *pflA*; Acetate kinase, *ackA*; NAD^+^-dependent D-lactate dehydrogenase, *ldhA*; Formate dehydrogenase, *fdxG*; NADH dehydrogenase, *ndh*; NADH dehydrogenase, *nqr*; L - Lactate dehydrogenase, *lldD*; D-Lactate dehydrogenase, *dld*; Cytochrome *bd* oxidase, *cydA, cydB*; DMSO reductase, *dmsA*; Nitrate reductase, *napA*; TMAO reductase, *torZ*; Nitrite reductase, *nrfA*; Fumarate reductase, *frdA*; malate dehydrogenase, *mdh*; PEP carboxylase, *ppc*; PEP carboxykinase, *pckA*; 2-oxoglutarate dehydrogenase, *sucAB*; Succinyl-CoA synthetase, *sucCD*; fumarate hydratase, *fumC*.

Early work indicated that at least 67% of *H. influenzae* proteins had homologs in *E. coli* and that the smaller genome size of *H. influenzae* was mostly caused by loss of paralogs (Tatusov et al., 1996). Reconstruction of the potential metabolic pathways present in the *H. influenzae* Rd genome (Edwards and Palsson, 1999) showed that this strain possesses a complete Embden-Meyerhof-Parnass (EMP) pathway (glycolysis) and pentose phosphate pathway (PPP) for glucose degradation, but lacks several enzymes of the TCA cycle (Figure 1). This latter property is typically associated with fermentative bacteria in which the TCA cycle is mostly used to produce precursors for biosynthesis. In *H. influenzae* the functional minimization of the TCA cycle is extreme with most enzymes of the oxidative branch being absent, including citrate synthase that mediates the initial condensation of acetyl-CoA and oxaloacetate (Figure 1).

Several other studies have used genomic and/or flux analysis approaches to date, where in most cases the only allowed products of *H. influenzae* metabolism were acetate, carbon dioxide and in some cases certain amino acids (Edwards and Palsson, 1999; Schilling and Palsson, 2000; Schilling et al., 2000; Papin et al., 2002). All of these studies appear to have aimed at determining the robustness of the *H. influenzae* metabolic network as well as the identification of stable flux states and the associated phenotypes. A combination of proteomic data and bioinformatics was used by Raghunathan et al. (2004) to study anaerobic and microaerophilic growth of *H. influenzae*, and led to the identification of formate and acetate as metabolic end-products using specific enzymatic detection protocols. Interestingly, the amounts of acetate and formate produced did not vary in response to changing oxygen availability (Raghunathan et al., 2004). Given the importance of central carbon metabolism in bacterial survival and colonization in the host we initiated a study of carbon metabolism in two strains of *H. influenzae*, a clinical isolate from a COPD patient (Hi2019) (Campagnari et al., 1987) and the reference strain Rd KW20 (HiRd) (Fleischmann et al., 1995) using a combination of targeted NMR-based metabolomics and qRT-PCR with the aim of understanding how this pathogen adapts its metabolism to varying oxygen availability.

## Methods

### Bacterial strains and growth conditions

*H. influenzae* Rd KW20 (Fleischmann et al., 1995) and 2019 (Campagnari et al., 1987) were grown at 37°C for 16–24 h using either brain heart infusion (BHI) broth (Difco) supplemented with hemin (10 μg/ml) and NAD (2 μg/ml) or chemically defined medium (CDM). 235 ml of CDM contained 191 ml RPMI 1640 (catalog no.: 11879-020; Life Technologies), 5.8 ml of 1M HEPES (pH 7.2–7.5, cat no.: 15630-080, Life Technologies), 2 ml of 100 mM MEM sodium pyruvate (catalog no.: 113600-070; Life Technologies), 10 ml uracil (2 mg/ml), 20 ml inosine (20 mg/ml), 2 ml NAD (1 mg/ml), 4 ml hemin (1 mg/ml), and 2.35 ml 1M glucose (final conc: 10 mM) as described in Coleman et al. (2003). BHI agar plates were supplemented with Levinthal's base (Alexander, 1965; Cooper et al., 2003). Cultures were grown under aerobic, microaerophilic, or anaerobic conditions as in (Cooper et al., 2003).

### Growth curves

Bacterial growth in liquid culture was determined in triplicate using CDM medium and three different oxygen concentrations (Cooper et al., 2003). Growth experiments used sterile 250 ml flasks (*aerobic* growth: 25 ml medium, shaking at 200 rpm, oxygen transfer coefficient (*k*_L_a) 87.4 h^−1^; *microaerophilic* growth: 150 ml medium, shaking at 200 rpm, oxygen transfer coefficient (*k*_L_a) 11.5 h^−1^) or completely filled 50 ml tubes. Main cultures were inoculated to an initial OD_600 nm_ of 0.15 (aerobic growth) or 0.05 (microaerophilic and anaerobic growth) from overnight cultures grown from glycerol stocks. Samples for OD_600 nm_ determination (0.1 ml) were taken every half hour for the first 4 h (aerobic growth) and every hour for microaerophilic and anaerobic growth for the first 8 h with a final reading for all cultures being taken after 24 h. All growth curves used triplicate samples for each growth condition tested on the day they were carried out, and repeat experiments (biological replicates) were conducted at least once for each growth condition and bacterial strain.

### Preparation of NMR samples and NMR spectroscopy

Samples for 1D ^1^H NMR were collected at three different time points, corresponding to early, mid and late exponential growth phases. Collection time points were selected based on growth curve experiments conducted in preparation for this work. To quantify metabolite concentrations in the medium 0.9 ml samples of *H. influenzae* culture were pelleted by centrifugation (3 min, RT, rcf: 20,000), the supernatants were removed to a fresh tube followed by the addition of azide to a final concentration of 0.01% from a 2% (w/v) stock and storage at −80°C. Samples for ^1^H NMR analysis had a volume of 550 μL and contained 500 μL of preserved culture supernatant, 10% D_2_O, 18 μM 2,2-dimethylsilapentane-5-sulfonic acid (DSS) and 18 μM 1,1-difluoro-1-trimethylsilanylphosphonic acid (DFTMP) in 5 mm NMR tubes. Proton NMR spectroscopy was performed on a Bruker AV500 500 MHz spectrometer (Bruker Biospin, Rheinstetten, Germany), with a 5 mm self-shielded z-gradient triple resonance probe. One-dimensional NOESY spectra were measured as previously described (Schirra et al., 2008) at 298 K with 128 scans at 32 k resolution with a spectral width of 14 ppm, using the noesy1prd pulse program (Bruker pulse program library). The water signal was suppressed with low-power continuous wave irradiation during the NOESY mixing time of 100 ms and the relaxation delay of 2.3 s. 1D spectra were processed using Topspin 3.0 (Bruker Biospin). Spectra were manually phased and baseline corrected, and chemical shifts were referenced to the DSS signal. Metabolites were identified by comparing putative identifications obtained using the Chenomx NMR Suite 7.1 (Chenomx Inc., Edmonton, Canada) with ^1^H chemical shifts from either 1 mM pure metabolite standards run separately, or by comparison with data from public databases such as BioMagResBank [www.bmrb.wisc.edu] or the Human Metabolome Database [www.hmdb.ca/]. NMR signals of metabolites of interest were manually integrated using Topspin 3.0 (Bruker Biospin) to obtain absolute integral intensities. Absolute integrals were calibrated in Chenomx NMR Suite 7.1 against the intensity of the DSS signal (18 μM) in the NMR spectrum of the uninoculated medium to obtain metabolite concentrations. Concentrations of metabolites not present in the uninoculated medium (labeled “Products” in Tables S5, S6) were calibrated relative to their concentration in the sample of strain 2019 grown for 4 h under microaerophilic conditions. NMR data of the metabolites of interest and the NMR signals used for concentration determination are listed in Table S1.

### Quantitative RT-PCR

Standard techniques were used throughout (Ausubel et al., 2005). RNA was isolated from 1.5 ml to 5 ml of *H. influenzae* cultures grown to an OD_600_ of 0.4. Culture samples were either pelleted by centrifugation followed by immediate freezing at −80°C or preserved using RNAprotect Bacteria Reagent (Qiagen) prior to freezing at −80°C. RNA was extracted using either the RNeasy Mini Kit (Qiagen) or the GE Healthcare Illustra RNAspin Mini Kit according to the manufacturer's instructions. RNA samples were tested for gDNA contamination using standard PCR (GoTaqgreen Mastermix, Promega) with RT_16S primers (Table S1). If no PCR product was observed after 34 cycles, samples were considered to be gDNA-free. RNA concentrations were determined using the Quant-it RNA kit (Life Technologies). cDNA was synthesized from 500 ng of RNA using the Superscript III Reverse Transcriptase (Life Technologies) and RNAsin RNase inhibitor (Promega). qRT-PCR (10 μl) reactions used diluted cDNA (1:10–1:100 000) as template, the SYBR green Mastermix (Applied Biosystems) and primers (Table S2) designed in Vector NTI Advance (Life Technologies) to produce PCR products of 120 bp. The 16S rDNA gene was used as the reference gene. For each individual experiment, qRT-PCR reactions were set up in triplicate (technical replicates) using an epMotion workstation (Eppendorf) and run on an ABI 7900 sequence detector. qRT-PCR experiments targetting all monitored genes were repeated at least three times for each strain with similar result being obtained. Data analysis and normalization was performed as in (Kappler et al., 2005).

### Enzyme assays

Cell cultures grown in BHI supplemented with 20 mM bicarbonate for 16–24 h at three different oxygen tensions were harvested by centrifugation (2370 × g, 4°C, 15 min). Cell pellets were stored at −20°C until further use. Cell extracts were prepared by resuspending the frozen pellets in 2 ml BugBuster Mastermix (Novagen). The samples were then incubated on a shaker at room temperature for 20 min followed by centrifugation (20238 × g, 5 min, RT). The supernatants were removed to clean tubes and were used for enzyme assays. DMSO reductase was assayed at 37°C as in (Jones and Garland, 1977) using a Hitachi UV3000-Spectrophotometer. The reaction was monitored at 600 nm using the oxidation of benzyl viologen (ε_600_ = 7.4 mM^−1^ cm^−1^) in the presence of either 17 mM DMSO or 5 mM MetSO (Lester and Demoss, 1971). Formate dehydrogenase activity was assayed according to (Enoch and Lester, 1982) by monitoring the reduction of dichlorophenolindophenol (DCPIP; ε_600_ = 21 mM^−1^ cm^−1^) (Lester and Demoss, 1971). All assay mixtures were degassed with nitrogen for 2 min before addition of the reductant and the cell extracts and used cuvettes sealed with rubber septa (Sigma Aldrich). Assays were conducted using at least two biological replicates (grown on different days) and at least three technical replicates (repeat assays) for each cell extract. Protein concentrations were determined using the Sigma-Aldrich BCA-1 protein determination kit.

## Results and discussion

### Central carbon metabolism in *H. influenzae*—pathways and energy generation

Analysis of available *H. influenzae* genomes clearly showed that the organization of the central carbon metabolic pathways (Figure 1) (Table S3) is highly conserved and confirmed results from previous work that had focused solely on the *H. influenzae* reference strain Rd (Fleischmann et al., 1995). All *H. influenzae* genomes encode enzymes for the EMP pathway (glycolysis), with the exception of glucokinase, and contain all enzymes of the PPP. Pyruvate, the main product of hexose degradation, can then be converted into a variety of other compounds (Figure 1). Acetyl-CoA can be produced using pyruvate dehydrogenase (encoded by *aceEF, lpdA*) or pyruvate formate lyase (*pflA*). Pyruvate can also be reduced to D-lactate by NAD-dependent lactate dehydrogenase (LdhA).

Of the TCA cycle enzymes only malate dehydrogenase, fumarate hydratase, succinyl CoA synthetase, and α-ketoglutarate dehydrogenase are present. All of these enzymes are important for the generation of biosynthetic precursors and the remnant TCA cycle is also linked to the respiratory chain via fumarate reductase. An exception to this is *H. influenzae* strain PittGG where the *frdA* gene encoding the large subunit of fumarate reductase contains a frameshift mutation, suggesting loss of function. Carbon units can enter this partial TCA cycle via the reaction of phosphenolpyruvate carboxykinase while acetyl-CoA produced by pyruvate dehydrogenase or pyruvate formate lyase can be converted to acetate via acetyl-P producing one ATP (Figure 1).

Incomplete TCA cycles are typically found in fermentative bacteria but in contrast to these, *H. influenzae* strains possess a versatile respiratory chain with five dehydrogenases (including NADH, lactate and formate dehydrogenases) transferring electrons into the menaquinone (MQ) pool, and at least six terminal reductases transferring the electrons to a variety of electron acceptors including oxygen, dimethylsulfoxide (DMSO) and various nitrogen compounds (Figure 1, Table S3). The components of this respiratory chain are almost completely conserved [exception: formate dehydrogenase and lactate dehydrogenase (LldD) in strain PittAA; *torZ* in strains 10810, 86-028NP, F3031, and 22.4-21]. This allows *H. influenzae* strains to use this versatile respiratory chain to easily re-oxidize the NADH produced by the partial oxidation of carbon sources under a variety of growth conditions.

We extended our analyses to include available genomes of other *Haemophilus* species (Table S4) which showed that virtually all *Haemophilus* genomes encode both glycolysis and PPP pathways. The presence of a partial TCA cycle is limited to *H. influenzae, Haemophilus parainfluenzae, Haemophilus haemolyticus*, and *Haemophilus ducreyi*, while most *Haemophilus parasuis* and *Haemophilus somnus* species appear to contain a complete TCA cycle. All *Haemophilus* species appeared to have reasonably versatile respiratory chains although some features such as the presence of the *dmsA* and *torZ* terminal reductases as well as three lactate dehydrogenases and especially the *lldD* and *dld* encoded enzymes, appear to be specific to *H. influenzae* (Tables S3, S4).

### Metabolic end products produced by *H. influenzae* strain RD (HiRd) change in response to changing oxygen tensions

**Figure 2:**
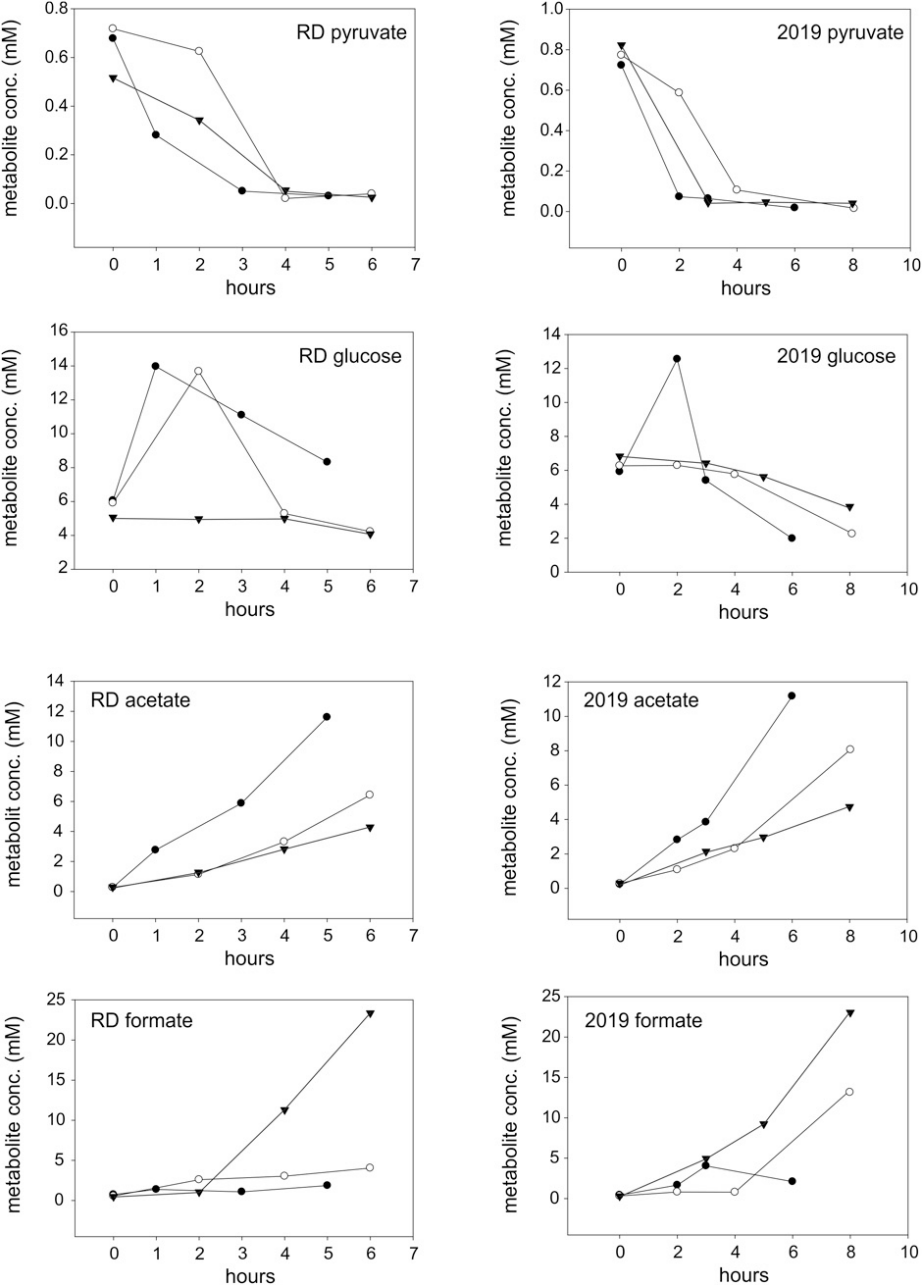
**Changes in metabolite concentrations in cultures of *H. influenzae* RD and 2019 grown under aerobic (closed circles), microaerophilic (open circles), and anaerobic (closed triangles) conditions.** The data shown is for two growth substrates, pyruvate and glucose (rows 1 and 2) and two key metabolites produced during growth, acetate, and formate (rows 3 and 4).

In keeping with the observation that *H. influenzae* only possess an incomplete TCA cycle, organic acids such as acetate and formate were the main products detected by ^1^H-NMR following aerobic, microaerophilic and anaerobic growth of *H. influenzae* in chemically defined medium (CDM) with glucose (Table 1). The two main carbon sources in the medium were differentially utilized. While pyruvate was quickly consumed (within 4 h following inoculation) glucose was initially produced by aerobic and microaerophilic cultures, presumably by gluconeogenesis, before being utilized in mid to late exponential phase (Figure 2). Acetate was the major end product during aerobic growth (Tables 1, S5), indicating that the dominant route of pyruvate catabolism was generation of acetyl-CoA and further conversion to acetate via acetyl phosphate.

**Table 1:** **Key metabolites detected in the growth medium of *H. influenzae* strain RD**.

**Metabolite**		**Initial conc (mM)**	**Aerobic (mM)**	**Microaerophilic (mM)**	**Anaerobic (mM)**
Substrates	Glucose	10	8.326	4.228	4.082
	Pyruvate	0.87	0.031	0.040	0.025
	Inosine	6.5	0.866	2.444	2.689
Products	Formate	n.d.^+^	1.86	4.05	23.36
	Acetate	n.d.^+^	11.61	6.43	4.28
	Hypoxanthine	n.d.^+^	3.53	1.75	0.701
	Glycerol	n.d.^+^	0.856	0.583	0.185
	Succinate	n.d.^+^	0.011	0.034	0.641
	Lactate	n.d.^+^	0.003	0.036	0.013

Samples were taken in late exponential phase. A list of minor metabolites detected is available in Table S5. ^+^not detected.

Under microaerobic conditions formate started to accumulate in addition to acetate (Tables 1, S5, Figure 2). This likely represents a switch in pyruvate metabolism from pyruvate dehydrogenase to an increased flux through pyruvate formate lyase (PFL), which during anaerobic growth of *H. influenzae* would lead to the observed accumulation of formate as the major end-product (Tables 1, S5).

Despite being typical products of bacterial “mixed acid” type fermentations, small organic acids such lactate and succinate were only produced in small amounts under all three conditions studied here (Tables 1, S5). Other metabolic endproducts were hypoxanthine (0.7–3.5 mM), probably resulting from the degradation of inosine, glycerol and glycine (4.38–1.04 mM, amounts decreasing with decreasing oxygen tensions) (Tables 1, S5).

Our results confirm that acetate and formate are the main metabolic end products of *H. influenzae* metabolism, but in contrast to a previous study that reported little or no variation in acetate (0.46–0.6 mM) and formate (0.29–0.3 mM) concentrations in microaerophilic and anaerobic *H. influenzae* cultures (Raghunathan et al., 2004), our data demonstrate a clear shift from acetate to formate as the main metabolic product in response to decreasing oxygen availability. The difference in the observed endproduct concentrations may be due to the use of different growth media. While this study used a chemically defined medium with glucose as the main carbon source, the study by Raghunathan et al. (2004) used a complex medium, Brain-Heart Infusion, that would likely have had a higher proportion of peptides and amino acids and a lower sugar contents than the tissue culture medium based CDM used in our work. While Brain-Heart Infusion is a standard medium for the cultivation of *H. influenzae*, the CDM formulation should more accurately reflect the conditions encountered by the bacteria when grown in contact with human cells. Preliminary experiments also (data not shown) indicated that the matrix of Brain-Heart Infusion is unsuitable for NMR analyses.

Our observations of changes in the relative amounts of metabolic endproducts produced by *H. influenzae* are consistent with constraints of the ability of the bacteria to maintain redox homeostasis in the absence of alternative external electron acceptors once oxygen becomes limiting. During aerobic growth when acetate is the main metabolic product NADH generated during glucose breakdown can be reoxidized by the respiratory chain and e.g., the cytochrome *bd* oxidase. Once oxygen becomes limiting, there is a switch to production of formate using PFL, which does not generate NADH. The alternative would be for pyruvate dehydrogenase to continue to be the major enzyme of pyruvate catabolism with NADH being consumed via lactate dehydrogenases and fumarate respiration, but the low levels of D-lactate and succinate produced suggest that these are minor pathways.

### Metabolic adaptation is supported by underlying changes in gene expression

19 genes encoding key enzymes involved in *H. influenzae* carbon metabolism and respiration were selected (Table S2) and changes in gene expression determined under the same conditions used for the NMR experiments.

**Figure 3:**
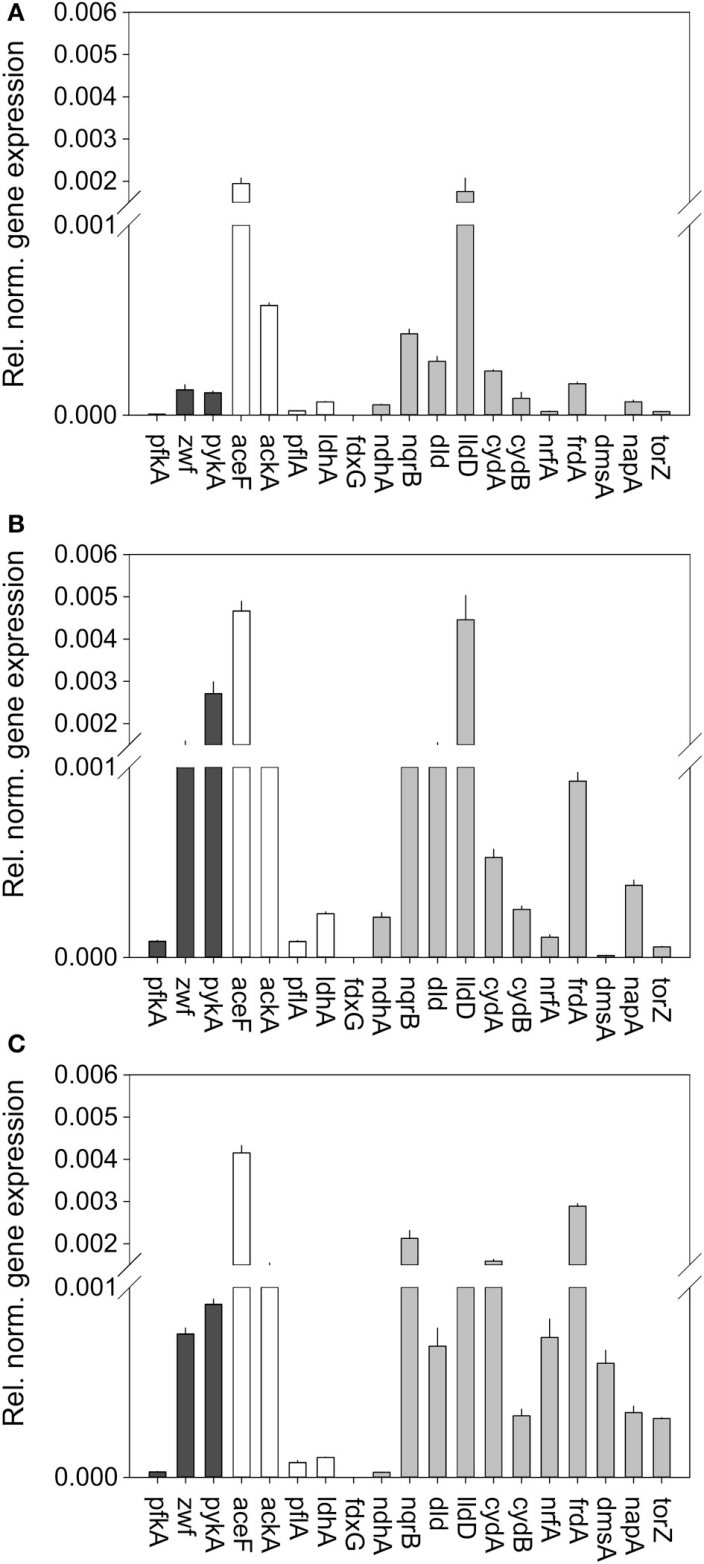
**Expression of genes involved in central carbon metabolism and respiration in *H. influenzae* RDKW20 for cultures grown under aerobic (A) microaerophilic (B) and anaerobic (C) conditions.** Black bars: Genes relevant for glucose catabolism, White bars: Genes relevant for pyruvate conversions, gray bars: genes relevant for respiratory metabolism. Abbreviations of gene names: Glucose 6-phosphate dehydrogenase, *zwf*; pyruvate dehydrogenase complex, *aceF*; Pyruvate formate lyase, *pflA*; Acetate kinase, *ackA*; NAD^+^-dependent D-lactate dehydrogenase, *ldhA*; Formate dehydrogenase, *fdxG*; NADH dehydrogenase, *ndh*; NADH dehydrogenase, *nqrB*; L-Lactate dehydrogenase, *lldD*; D-Lactate dehydrogenase, *dld*; Cytochrome bd oxidase, *cydA, cydB*; DMSO reductase, *dmsA*; Nitrate reductase, *napA*; TMAO reductase, *torZ*; Nitrite reductase, *nrfA*; Fumarate reductase, *frdA*.

Interestingly, under all growth conditions tested high levels of pyruvate dehydrogenase (*aceF*), acetate kinase (*ackA*), and the respiratory L-lactate dehydrogenase (*lldD*) were detected, indicating key roles for these enzymes in *H. influenzae* metabolism (Figure 3). In fact, HiRd is capable of growth using lactate as a carbon source (data not shown), and the LldD enzyme could be important under those conditions. It has also been suggested that LldD might be important during invasive disease as lactate is commonly present in blood (Wong et al., 2007).

Based on the metabolic model (Figure 1) the initial degradation of glucose in *H. influenzae* would occur using either the PPP or glycolysis pathways, and interestingly both 6-phosphofructokinase (*pfkA*) and glucose-6-phosphate 1- dehydrogenase (*zwf*) which represent glycolysis and PPP, respectively, as well as pyruvate kinase (*pykA)* were expressed most highly under microaerophilic and anaerobic conditions. However, relative expression levels of *zwf* (and *pykA*) were much higher than those of *pfkA* (16–27 fold and 24–31 fold, respectively) (Figure 3) suggesting that HiRd uses mainly the PPP for glucose metabolism. Two enzymes that would use the product of glucose breakdown, pyruvate, pyruvate-formate lyase (*pflA*), and the NAD^+^-dependent LdhA lactate dehydrogenase both showed increased expression levels during microaerophilic and anaerobic growth.

Of the respiratory chain enzymes all five respiratory dehydrogenases were most highly expressed under microaerophilic conditions, with the two lactate dehydrogenases, LldD and Dld and the sodium-translocating NADH dehydrogenase (Nqr) being expressed at high levels, while formate dehydrogenase and the non-energy conserving NdhA NADH dehydrogenase were expressed at low levels at all times (Figure 3). Expression of *nqr* genes was also high under anaerobic conditions.

In contrast, for all six terminal reductases including fumarate reductase (*frdA*) and the cytochrome *bd* oxidase relative expression levels were highest under anaerobic conditions. The expression pattern of the cytochrome bd oxidase is thus similar to what has been seen in *E. coli* (Cotter et al., 1990). The gene encoding the NapA nitrate reductase was the only one that showed similar expression levels under microaerophilic and anaerobic conditions, while genes encoding sulfoxide reductase related enzymes (*dmsA, torZ*) and nitrite reductase (*nrf*) were only expressed at significant levels under anaerobic conditions. This is consistent with the role of nitrate and sulfoxide reductases as alternative terminal reductases that has been described for a variety of bacteria (Richardson et al., 2001; McCrindle et al., 2005).

A number of the genes studied here have been previously identified as being under the control of major regulators of aerobic/anaerobic transitions in bacterial metabolism namely FNR (relevant target genes: *dmsA, napA, nrfA*) and the ArcAB two component system (relevant target genes: *fdx, frd, lldD, ndh*) (De Souza-Hart et al., 2003; Wong et al., 2007; Harrington et al., 2009). While the three FNR regulated genes studied here showed the expected increase in gene expression under anaerobic conditions already reported previously (Harrington et al., 2009), the case for the ArcA regulated genes is not so clear cut. A study using a proteomic approach observed and Arc regulon of ~40 proteins in H. influenzae, and identified three of these using mass spectrometry, namely the Fdx formate dehydrogenase and the Frd fumarate reductase, both of which were induced under anaerobic conditions, and the lldD lactate dehydrogenase that was subject to Arc-based repression under both aerobic and anaerobic growth conditions (De Souza-Hart et al., 2003). These observations largely agree with our data reported above, where *fdx* and *frd* are expressed at higher levels when oxygen becomes limiting, although for *fdx* we observed peak expression and activity under microaerophilic rather than fully anaerobic conditions. A study monitoring gene expression changes in an H. influenzae *arcA* mutant under anaerobic conditions identified a total of 23 genes controlled by ArcA which included *fdx, lldD*, and *ndh* (Wong et al., 2007). All three of these genes were reported to be negatively regulated by ArcA under anaerobic conditions which is in agreement with our gene expression data (Figure 3). The *frd* fumarate reductase was not identified as a target of Arc–regulation by (Wong et al., 2007). Data from our work and these previous studies may indicate that regulation of some enzyme such as the Fdx formate dehydrogenase is complex and may involve more than one regulator.

In summary, our data presented above indicate that *H. influenzae* metabolism is likely adapted to conditions where low levels of oxygen are present as most of the available respiratory pathways, including oxygen based respiration, are highly expressed under these conditions. In *H. influenzae* RD there also appears to be a preference for glucose degradation via the PPP rather than via glycolysis, which allows an increased production of NADPH and pentose sugars for the synthesis of nucleotides.

**Figure 4:**
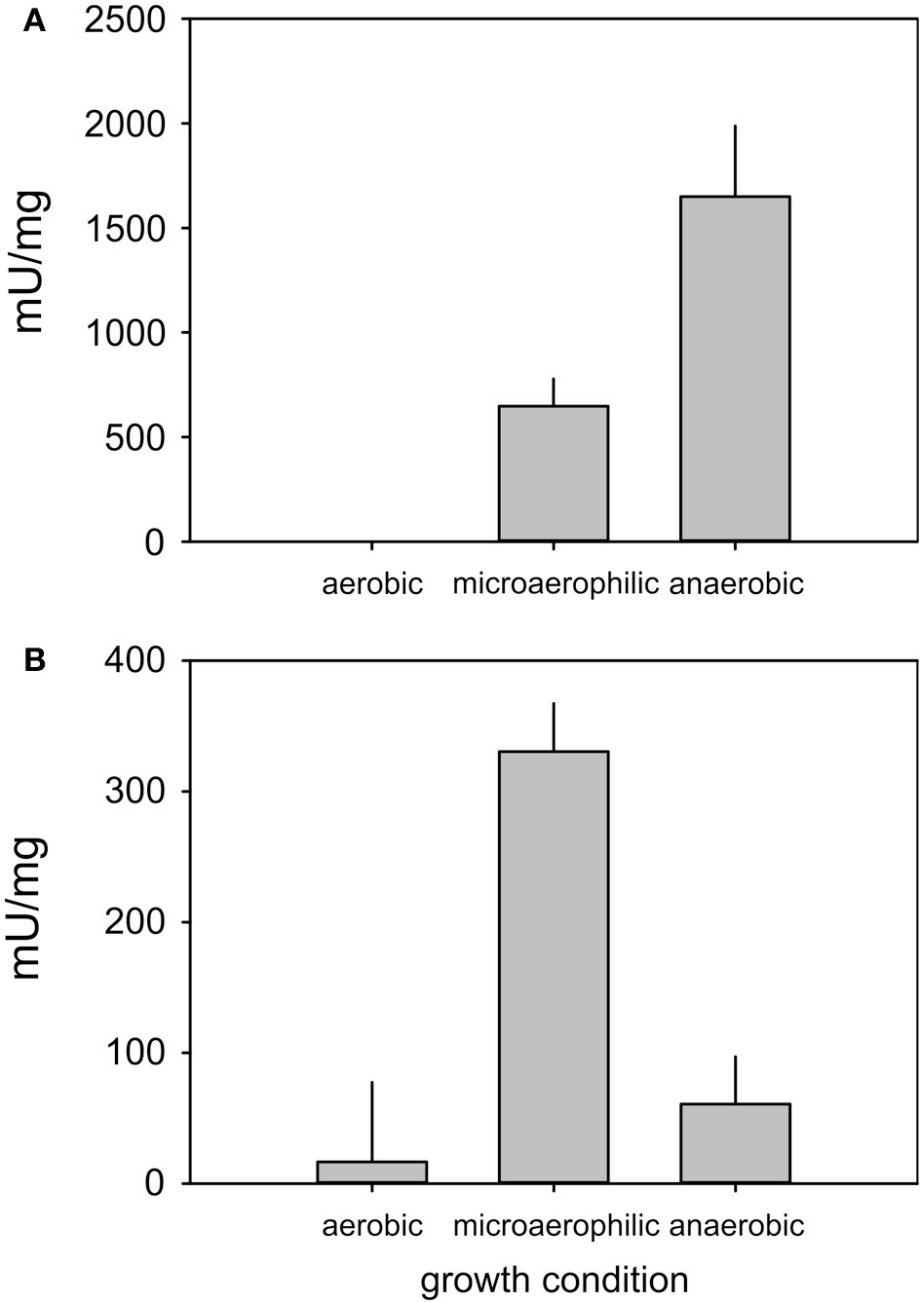
**Enzyme activities of DMSO reductase (A) and Formate dehydrogenase (B) in *H. influenzae* RD under aerobic, microaerophilic, and anaerobic growth conditions.** Assays were conducted with at least three repetitions, the experimental error is reported as standard error of the mean.

As expression of the formate dehydrogenase encoding genes appeared to be extremely low (Figure 3) we conducted enzyme assays for formate dehydrogenase and, as a control, DMSO reductase to confirm the qRT-PCR results (Figure 4). In keeping with the qPCR results the highest level of formate dehydrogenase activity was detected in cell grown under microaerophilic conditions (~350 mU/mg), while DMSO reductase activity was highest in extracts made from anaerobically grown cells (~1500 mU/mg), which is in keeping with the known FNR-based reglation of the *dmsA* promoter (Harrington et al., 2009). In *E. coli*, both enzymes are known to be active under conditions when oxygen concentrations are low (Gladyshev et al., 1996; McNicholas et al., 1997).

The high expression levels of formate dehydrogenase under microaerophilic conditions and the high levels of formate detected as a metabolic endproduct during anaerobic growth suggest a connection between the relative activities of pyruvate formate lyase (*pflA*) and formate dehydrogenase (*fdxG)* which produce and degrade formate, respectively. The presence of formate dehydrogenase activity under microaerophilic growth may lead to an underestimation of the actual flux of carbon via PFL (Figure 2, Tables 1, S5) as the formate produced would be further degraded to carbon dioxide. Formate dehydrogenase is an energy-conserving Mo enzyme in the respiratory chain and in combination with the cytochrome *bd* oxidase can generate a proton motive force. The results infer that under microaerobic conditions respiration with formate as an electron donor and oxygen as electron acceptor allows additional energy conservation via an increased proton motive force. Under anaerobic conditions and in the absence of other external respiratory electron acceptors formate cannot be further oxidized since *H. influenzae* does not possess formate-hydrogen lyase.

### Do all *H. influenzae* strains show the same pattern of metabolic adaptation?

While being widely used in research, the Rd reference strain has been in laboratory culture for decades, and we therefore extended our analyses to a non-typeable strain of *H. influenzae*, strain 2019 (Hi2019) which is an isolate from a patient suffering from COPD (Campagnari et al., 1987).

Overall, the spectrum of metabolic end products was the same in both *H. influenzae* strains, but there were subtle changes in the amounts as well as types of minor products present (Tables 2, S6), and in the consumption of substrates. For example, in Hi2019 depletion of pyruvate was faster that in HiRD, and the final concentrations of glucose present in the growth medium were lower than in HiRD indicating a more complete utilization of the carbon source (Figure 2). The key metabolites produced by Hi2019 were similar to those observed previously, although larger amounts of formate (13.16 vs. 4.05 mM for HiRD) were produced under microaerophilic conditions (Tables 2, S6). No significant amounts of glycerol or glycine were present in the growth medium following growth of HI2019 although both metabolites were produced transiently during growth of the strain, with glycine concentrations reaching a maximal concentration of ~1.2 mM (data not shown).

**Table 2:** **Key metabolites detected in the growth medium of *H. influenzae* 2019**.

**Metabolite**		**Initial conc. (mM)**	**Aerobic (mM)**	**Microaerophilic (mM)**	**Anaerobic (mM)**
Substrates	Glucose	10	1.98	2.26	3.864
	Pyruvate	0.87	0.018	0.015	0.041
	Inosine	6.5	2.60	3.043	3.347
Products	Formate	n.d.^+^	2.09	13.16	23.04
	Acetate	n.d.^+^	11.17	8.06	4.74
	Hypoxanthine	n.d.^+^	1.63	1.53	1.242
	Glycerol	n.d.^+^	0.046	0.04	0.032
	Succinate	n.d.^+^	0.007	0.401	1.009
	Lactate	n.d.^+^	0.069	0.098	0.456

Samples were taken in late exponential phase. A list of minor metabolites detected is available in Table S6. ^+^not detected.

**Figure 5:**
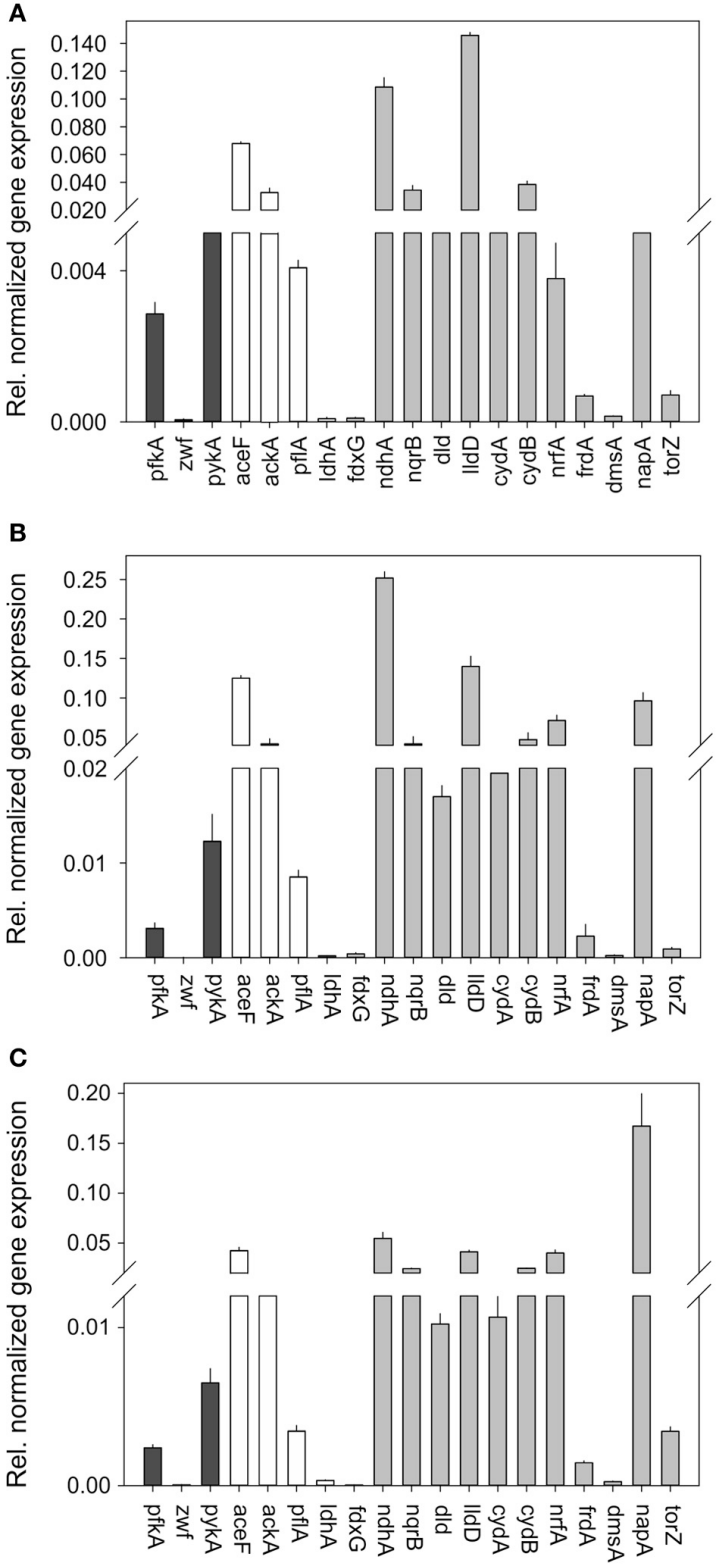
**Expression of genes involved in central carbon metabolism and respiration in *H. influenzae* 2019 for cultures grown under aerobic (A) microaerophilic (B) and anaerobic (C) conditions.** Black bars: Genes relevant for glucose catabolism, White bars: Genes relevant for pyruvate conversions, gray bars: genes relevant for respiratory metabolism. Abbreviations of gene names: Glucose 6-phosphate dehydrogenase, *zwf*; pyruvate dehydrogenase complex, *aceF*; Pyruvate formate lyase, *pflA*; Acetate kinase, *ackA*; NAD^+^-dependent D-lactate dehydrogenase, *ldhA*; Formate dehydrogenase, *fdxG*; NADH dehydrogenase, *ndh*; NADH dehydrogenase, *nqrB*; L-Lactate dehydrogenase, *lldD*; D-Lactate dehydrogenase, *dld*; Cytochrome bd oxidase, *cydA, cydB*; DMSO reductase, *dmsA*; Nitrate reductase, *napA*; TMAO reductase, *torZ*; Nitrite reductase, *nrfA*; Fumarate reductase^‡^, *frdA*.

We then conducted studies of gene expression in *H. influenzae* 2019 using the same set of genes used for HiRd previously (Figure 5). Most of the gene expression patterns were similar to HiRD, some significant differences were also observed. The expression levels of *pfkA* (glycolysis) exceeded those of *zwf* (PPP) between 50 and 89 times, indicating that in Hi2019 glycolysis is the main pathway for glucose catabolism. The *ndhA* gene was only expressed at low levels in HiRD but is always expressed at high levels in Hi2019. This is interesting as the NdhA protein oxidizes NADH without energy conservation via proton translocation which would have implications for the energy balance. Expression of the second NADH dehydrogenase complex (*nqrB*) appeared to follow a similar expression pattern as in HiRD.

Of the other respiratory chain components two of the terminal reductases showed clearly changed gene expression patterns in Hi2019, namely the *nrfA* (nitrite reductase) and the *napA* (nitrate reductase) genes both of which seemed to be expressed at much higher levels than in HiRd relative to other enzymes of the respiratory chain.

These data lead us to propose that despite their nearly identical genetic makeup glucose metabolism in HiRd and Hi2019 uses different main pathways. In HiRd the dominant catabolic route is propose to involve the PPP which generates NADPH (used in biosynthesis rather than being used in the respiratory chain) and glyceraldehyde 3-P. In contrast, Hi2019 appears to use glycolysis as the main pathway, where an additional molecule of ATP is consumed during activation of glucose. Hi2019 may also have a greater dependence on the respiratory chain, especially NADH dehydrogenase and nitrate reductase.

The data presented above also clearly show that despite the differences in the regulation and utilization of different respiratory chain complexes, both strains of *H. influenzae* use a type of metabolism that could be called “respiration-assisted” fermentation, i.e., a fermentative type of metabolism in which problems with redox homeostasis due to overreduction of the cellular NAD^+^ pool which can be caused by purely fermentative growth can be alleviated through the activities of the respiratory enzymes.

Despite the presence of a respiratory chain *H. influenzae* metabolism is configured so that simple chain carboxylic acids will always be the main product regardless of the presence or absence of oxygen. As a result the regulatory mechanisms at play in *H. influenzae* can be expected to be quite different from those found e.g., in *E. coli* and other bacteria capable of aerobic respiration in the presence of a complete TCA cycle. Thus, the switch from acetate as the main product to formate in *H. influenzae* may be due to a change in the main route of pyruvate metabolism, the underlying mechanisms are likely unrelated to the “acetate switch” described for *E. coli* (Wolfe, 2005).

An interesting feature of the *H. influenzae* respiratory chain is that it contains a surprisingly large number of components not associated with energy conservation via a proton gradient (e.g., NdhA, Nqr, Nap, TorZ) which may also represent an adaptation to the host environment. Differential regulation of respiratory chain composition and pathways associated with energy generation have been shown to be associated with virulence in *Escherichia coli* infecting the urinary tract (Alteri et al., 2011) and the possibility that similar mechanisms exist in *H. influenzae* should be investigated in the future. While a metabolic arrangement that has low efficiency in energy conservation may appear unusual, there is some evidence for *Salmonella enterica* sv typhimurium that an incomplete TCA cycle enhances survival of the bacteria in macrophages (Bowden et al., 2010) and similar effects may be relevant in *H. influenzae* but will require further investigation. Interestingly, for *H. influenzae* serotype b it has been reported that a loss of either pyruvate dehydrogenase (*aceEF*) or α-ketoglutarate dehydrogenase (*sucAB*) leads to a significant attenuation of virulence in a mouse model of systemic disease (Herbert et al., 2003). This underlines the importance of metabolism and metabolic enzymes for *H. influenzae* pathogenesis and survival *in vivo*.

## Author contributions

Dk Seti Maimonah Pg Othman carried out the majority of the experimental work, Horst Schirra collected the NMR data and advised on appropriate data analysis, Alastair G. McEwan and Ulrike Kappler were responsible for the study design and experimental work. All authors contributed to the writing of the manuscript.

### Conflict of interest statement

The authors declare that the research was conducted in the absence of any commercial or financial relationships that could be construed as a potential conflict of interest.

## Supplementary material

Table S1 **Chemical shift multiplicity and signal regions used for metabolite identification and quantification**.

Table S2 **Representative genes involved in *H.influenzae* central carbon and respiratory metabolism and relevant qRT-PCR primers used in this work**.

Table S3 **Presence of central carbon metabolism genes in genomes of *H. influenzae* strains**.

Table S4 **Presence of genes involved in central carbon metabolism in genomes of *Haemophilus sp.* other than *H. influenzae***.

Table S5 ***H. influenzae* RD NMR metabolites detected in the growth medium of samples taken in late exponential growth phase.** n.d.—not detected.

Table S6 ***H. influenzae* 2019 NMR metabolites detected in the growth medium of samples taken in late exponential growth phase.** n.d.—not detected.
