# Supplementary material for: Metabolic versatility in Haemophilus influenzae: a metabolomic and genomic analysis
Source: Front Microbiol. 2014 Mar 4;5:69. doi: 10.3389/fmicb.2014.00069 (PMC3941224; doi:10.3389/fmicb.2014.00069)
Supplement: Table S1 — Chemical shift multiplicity and signal regions used for metabolite identification and quantification. [file DataSheet1.ZIP › 75817_Kappler_Suppl_Table_2.DOCX]

**Table S2: Representative genes involved in NTHi central carbon and respiratory metabolism and relevant qRT-PCR primers used in this work**

| Role in metabolism | Function | Gene | qPCR- primer sequences |
| --- | --- | --- | --- |
| Glycolysis | 6-phosphofructokinase | *pfkA* | **F:** CGATATATTCACAACCGCCAG  **R:** TCGACCGTTTACGTGATACAT |
|  | Pyruvate kinase | *pykA* | **F:** TTGCAGACATCGCAACAGATT  **R:** CAGTGGCAGCAATGGCTAG |
| Pentose phosphate pathway | Glucose 6-phosphate dehydrogenase | *zwf* | **F:** CTCTTCCACGCCAATAGATTC  **R:** GAAACCGTTCAAAACTTGCTC |
| Pyruvate metabolism | Part of pyruvate dehydrogenase complex | *aceF* | **F:** AGCCAGCACCATTTGCTACAC  **R:** GTCGTAAAGGTCGTATCGTTA |
|  | Pyruvate formate lyase | *pflA* | **F:** CTCTTTACAAGCACGGAACC  **R:** AAAGAAGTCGTGACTTATCGC |
|  | Acetate kinase | *ackA* | **F:** CCAATTAAGTGAGCAGGGTTG  **R:** TCGTATCGTTCACGGTGGCG |
|  | D-Lactate dehydrogenase | *ldhA* | **F:** CTGGCGTTGCTGGGCAATGA  **R:** TCTTGGCGTACGATCCTTTCA |
| Electron transport chain | Formate dehydrogenase | *fdxG* | **F:** GTGGCGGTATTAACGCATTAC  **R:** CGTAAGAGGTATCTCTATCGT |
|  | NADH dehydrogenase | *ndh* | **F:** GGAAATGGGCACCAATGTGCA  **R:** CGAACCCCTGCTGCCCAAAC |
|  | NADH dehydrogenase | *nqrB* | **F:** TGCAACTTGGGGCTCTAAAAT  **R:** ACTTCGTGACCACGAACCAC |
|  | L-Lactate dehydrogenase^+^ | *lldD* | **F:** ATCATACGCCCAGAATGGGTG  **R:** ACGGTCGATATGCCTACTCCA |
|  | D-Lactate dehydrogenase^+^ | *dld* | **F:** AGCGGAAAGGTGTCTAAACGA  **R:** TCGTCAAGTTGATGAAGGCTC |
|  | Cytochrome bd oxidase | *cydA* | **F:** AACCAACCACACTCAATGGCA  **R:** ACTTACATTCGGCGCATTTGT |
|  |  | *cydB* | **F:** CGTGAGTGACAAGCATTGATA  **R:** GCGTTCCATTCCATTTTAACG |
|  | DMSO reductase | *dmsA* | **F:** CAAGCACGAACCTGATGATCA  **R:** AGTAAACTGTGGTAGCCGTTG |
|  | Nitrate reductase | *napA* | **F:** TGGTCGCGGTCACGGTCA  **R:** GGATCATAGCCTTCACGGTAA |
|  | TMAO reductase | *torZ* | **F:** AACAAACGGGTTACCACCTGC  **R:** CCATTAGCGCGTATTGCTGAT |
|  | Nitrite reductase | *nrfA* | **F:** GCCGTGTAAACCTGAAGATGC  **R:** ATCCCTGTTCGTCCAGTTGTT |
|  | Fumarate reductase^‡^ | *frdA* | **F:** GCCGTGTAAACCTGAAGATGC  **R:** ATCCCTGTTCGTCCAGTTGTT |
