# Supplementary material for: Metabolic versatility in Haemophilus influenzae: a metabolomic and genomic analysis
Source: Front Microbiol. 2014 Mar 4;5:69. doi: 10.3389/fmicb.2014.00069 (PMC3941224; doi:10.3389/fmicb.2014.00069)
Supplement: Table S1 — Chemical shift multiplicity and signal regions used for metabolite identification and quantification. [file DataSheet1.ZIP › 75817_Kappler_Suppl_Table_3 and 4.DOCX]

**Table S3** Presence of central carbon metabolism genes in genomes of *H. influenzae* strains

| Metabolic function | glucose deg. | | | | pyruvate conversion | | | | | respiratory chain components | | | | | | | | | | | | | TCA cycle | | | |
| --- | --- | --- | --- | --- | --- | --- | --- | --- | --- | --- | --- | --- | --- | --- | --- | --- | --- | --- | --- | --- | --- | --- | --- | --- | --- | --- |
| Strain/Gene | *pfkA* | *zwf* | *pykA* | *aceF* | | *ackA* | *pflA* | *ldhA* | *fdxG* | | *ndh* | *nqrB* | *dld* | *lldD* | *cydA* | *cydB* | *frdA* | *dmsA* | *torZ* | *napA* | *nrfA* | *sucB* | | *sucC* | *fumC* | *mdh* |
| **Rd KW20** | √ | √ | √ | √ | | √ | √ | √ | √ | | √ | √ | √ | √ | √ | √ | √ | √ | √ | √ | √ | √ | | √ | √ | √ |
| **F3047** | √ | √ | √ | √ | | √ | √ | √ | √ | | √ | √ | √ | √ | √ | √ | √ | √ | √ | √ | √ | √ | | √ | √ | √ |
| **10810** | √ | √ | √ | √ | | √ | √ | √ | √ | | √ | √ | √ | √ | √ | √ | √ | √ |  | √ | √ | √ | | √ | √ | √ |
| **86-028NP** | √ | √ | √ | √ | | √ | √ | √ | √ | | √ | √ | √ | √ | √ | √ | √ | √ |  | √ | √ | √ | | √ | √ | √ |
| **F3031** | √ | √ | √ | √ | | √ | √ | √ | √ | | √ | √ | √ | √ | √ | √ | √ | √ |  | √ | √ | √ | | √ | √ | √ |
| **PittEE** | √ | √ | √ | √ | | √ | √ | √ | √ | | √ | √ | √ | √ | √ | √ | √ | √ | √ | √ | √ | √ | | √ | √ | √ |
| **PittGG** | √ | √ | √ | √ | | √ | √ | √ | √ | | √ | √ | √ | √ | √ | √ | √ | √ | √ | √ | √ | √ | | √ | √ | √ |
| **R2846** | √ | √ | √ | √ | | √ | √ | √ | √ | | √ | √ | √ | √ | √ | √ | √ | √ | √ | √ | √ | √ | | √ | √ | √ |
| **R2866** | √ | √ | √ | √ | | √ | √ | √ | √ | | √ | √ | √ | √ | √ | √ | √ | √ | √ | √ | √ | √ | | √ | √ | √ |
| **2019** | √ | √ | √ | √ | | √ | √ | √ | √ | | √ | √ | √ | √ | √ | √ | √ | √ | √ | √ | √ | √ | | √ | √ | √ |
| 21.1-21 | √ | √ | √ | √ | | √ | √ | √ | √ | | √ | √ | √ | √ | √ | √ | √ | √ | √ | √ | √ | √ | | √ | √ | √ |
| 22.4-21 | √ | √ | √ | √ | | √ | √ | √ | √ | | √ | √ | √ | √ | √ | √ | √ | √ |  | √ | √ | √ | | √ | √ | √ |
| 3655 | √ | √ | √ | √ | | √ | √ | √ | √ | | √ | √ | √ | √ | √ | √ | √ | √ | √ | √ | √ | √ | | √ | √ | √ |
| 6P18H1 | √ | √ | √ | √ | | √ | √ | √ | √ | | √ | √ | √ | √ | √ | √ | √ | √ | √ | √ | √ | √ | | √ | √ | √ |
| 7P49H1 | √ | √ | √ | √ | | √ | √ | √ | √ | | √ | √ | √ | √ | √ | √ | √ | √ | √ | √ | √ | √ | | √ | √ | √ |
| NT127 | √ | √ | √ | √ | | √ | √ | √ | √ | | √ | √ | √ | √ | √ | √ | √ | √ | √ | √ | √ | √ | | √ | √ | √ |
| PittAA | √ | √ | √ | √ | | √ | √ | √ |  | | √ | √ | √ |  | √ | √ | √ | √ | √ | √ | √ | √ | | √ | √ | √ |
| PittHH | √ | √ | √ | √ | | √ | √ | √ | √ | | √ | √ | √ | √ | √ | √ | √ | √ | √ | √ | √ | √ | | √ | √ | √ |
| PittII | √ | √ | √ | √ | | √ | √ | √ | √ | | √ | √ | √ | √ | √ | √ | √ | √ | √ | √ | √ | √ | | √ | √ | √ |
| R3021 | √ | √ | √ | √ | | √ | √ | √ | √ | | √ | √ | √ | √ | √ | √ | √ | √ | √ | √ | √ | √ | | √ | √ | √ |
| RdAW | √ | √ | √ | √ | | √ | √ | √ | √ | | √ | √ | √ | √ | √ | √ | √ | √ | √ | √ | √ | √ | | √ | √ | √ |

Gene abbreviations used: pyruvate dehydrogenase complex, *aceF*; Pyruvate formate lyase, *pflA*; Acetate kinase , *ackA*; Lactate dehydrogenase, *ldhA*; Formate dehydrogenase, *fdxG*; NADH dehydrogenase, *ndh*; NADH dehydrogenase, *nqr*; L-Lactate dehydrogenase^+^, *lldD*; D-Lactate dehydrogenase^+^, *dld*; Cytochrome *bd* oxidase, *cydA*, *cydB*; DMSO reductase, *dmsA*; Nitrate reductase, *napA*; TMAO reductase, *torZ*; Nitrite reductase, *nrfA*; Fumarate reductase^‡^, *frdA;*  isocitrate dehydrogenase, *idh*; citrate synthase, *gltA*; aconitase, *acn*; succinate ddehdyrogenase *sdh*.

**Table S4** Presence of genes involved in central carbon metabolism in genomes of *Haemophilus sp.* other than *H. influenzae*

|  | glucose deg. | | | pyruvate conversion | | | | respiratory chain components | | | | | | | | | | | | TCA cycle | | | | | | | |
| --- | --- | --- | --- | --- | --- | --- | --- | --- | --- | --- | --- | --- | --- | --- | --- | --- | --- | --- | --- | --- | --- | --- | --- | --- | --- | --- | --- |
| Strain/Gene | *pfkA* | *zwf* | *pykA* | *aceF* | *ackA* | *pflA* | *ldhA* | *fdxG* | *ndh* | *nqrB* | *dld* | *lldD* | *cydA* | *cydB* | *frdA* | *dmsA* | *torZ* | *napA* | *nrfA* | *sucB* | *sucC* | *fumC* | *mdh* | *idh* | *gltA* | *can* | *sdh* |
| ***HPI T3T1*** | *√* | *√* | *√* | *√* | *√* | *√* |  | *√* | *√* | *√* |  |  | *√* | *√* | *√* |  |  | *√* | *√* | *√* | *√* | *√* | *√* |  |  |  |  |
| *HPI ATCC 33392* | *√* | *√* | *√* | *√* | *√* | *√* |  | *√* | *√* | *√* |  | *√* | *√* | *√* | *√* |  |  | *√* | *√* | *√* | *√* | *√* | *√* |  |  |  |  |
| *HPI HK2019* | *√* | *√* | *√* | *√* | *√* | *√* |  | *√* | *√* | *√* |  | *√* | *√* | *√* | *√* |  | *√* | *√* | *√* | *√* | *√* | *√* | *√* |  |  |  |  |
| *HPI HK262* | *√* | *√* | *√* | *√* | *√* | *√* |  | *√* | *√* | *√* |  | *√* | *√* | *√* | *√* |  | *√* | *√* | *√* | *√* | *√* | *√* | *√* |  |  |  |  |
| **HD 35000HP** | *√* | *√* | *√* | *√* | *√* | *√* |  |  | *√* | *√* |  | *√* | *√* | *√* | *√* |  | *√* | *√* | *√* | *√* |  | *√* | *√* |  |  |  |  |
| *HH M19501* | √ | √ | √ | √ | √ | √ | √ | √ | √ | √ |  |  | √ | √ | √ | √ |  | √ | √ | √ | √ | √ | √ |  |  |  |  |
| *HH HK386* | √ | √ | √ | √ | √ | √ | √ | √ | √ | √ |  |  | √ | √ | √ |  |  | √ | √ | √ | √ | √ | √ |  |  |  |  |
| *HH M19107* | √ | √ | √ | √ | √ | √ | √ | √ | √ | √ |  |  | √ | √ | √ |  |  | √ | √ | √ | √ | √ | √ |  |  |  |  |
| *HH M21127* | √ | √ | √ | √ | √ | √ | √ | √ | √ | √ |  |  | √ | √ | √ |  |  | √ | √ | √ |  | √ | √ |  |  |  |  |
| *HH M21639* | √ | √ | √ | √ | √ | √ | √ | √ | √ | √ |  | √ | √ | √ | √ | √ | √ | √ | √ | √ | √ | √ | √ |  |  |  |  |
| *HH M21621* | √ | √ | √ | √ | √ | √ | √ | √ | √ | √ |  |  | √ | √ | √ |  |  | √ | √ | √ | √ | √ | √ |  |  |  |  |
| **HP ZJ0906** | √ | √ | √ | √ | √ | √ | √ | √ | √ | √ |  |  | √ | √ | √ |  |  | √ | √ | √ | √ | √ | √ | √ | √ | √ |  |
| **HP SH0165** | √ | √ | √ | √ | √ | √ | √ | √ | √ | √ | √ |  | √ | √ | √ |  |  | √ | √ | √ | √ | √ | √ | √ | √ | √ |  |
| HP 12939 | √ | √ | √ | √ | √ | √ | √ | √ | √ | √ |  |  | √ | √ | √ |  |  | √ | √ | √ | √ | √ | √ |  | √ | √ |  |
| HP 174 | √ |  | √ |  | √ |  |  | √ |  | √ |  |  | √ | √ |  |  |  | √ | √ | √ | √ |  |  | √ | √ |  |  |
| HP 29755 | √ |  | √ | √ |  | √ | √ | √ |  |  |  |  | √ | √ | √ |  |  |  |  | √ |  | √ | √ | √ | √ | √ |  |
| HP SW140 | √ | √ | √ | √ |  |  |  | √ |  |  |  |  | √ | √ | √ |  |  | √ |  | √ | √ | √ | √ |  |  |  |  |
| HP Nagasaki | √ | √ | √ | √ | √ | √ |  | √ | √ | √ | √ |  | √ |  | √ |  |  | √ | √ | √ | √ | √ | √ | √ | √ | √ |  |
| HP GX033 | √ | √ | √ | √ | √ | √ | √ | √ | √ | √ |  |  | √ |  | √ |  |  |  | √ | √ | √ | √ | √ | √ | √ | √ |  |
| HP MN-H | √ | √ | √ |  |  | √ | √ | √ |  | √ |  |  | √ | √ | √ |  |  | √ | √ | √ | √ | √ |  |  |  |  |  |
| HP H465 | √ | √ | √ |  |  | √ |  | √ | √ |  | √ |  | √ | √ | √ |  |  |  |  | √ | √ | √ | √ | √ | √ | √ |  |
| HP 84-15995 | √ | √ | √ | √ | √ | √ | √ | √ |  | √ |  |  | √ | √ | √ |  |  | √ |  | √ | √ | √ | √ | √ | √ | √ |  |
| HP SW114 | √ | √ | √ | √ | √ | √ | √ |  | √ | √ |  |  | √ | √ | √ |  |  | √ | √ | √ | √ | √ | √ | √ | √ | √ |  |
| HP D74 | √ | √ | √ | √ | √ | √ | √ |  | √ | √ | √ |  | √ | √ | √ |  |  | √ | √ | √ | √ |  |  | √ | √ | √ |  |
| *HPH HK385* | √ | √ | √ | √ | √ | √ | √ | √ | √ | √ | √ | √ | √ | √ | √ |  | √ | √ | √ | √ | √ | √ | √ |  |  |  |  |
| HA ATCC 11116 | √ | √ | √ | √ | √ | √ | √ | √ | √ | √ | √ | √ | √ | √ | √ | √ |  | √ | √ | √ | √ | √ | √ |  |  |  |  |
| *HPT HK 85* | √ | √ | √ | √ | √ | √ | √ | √ | √ | √ | √ |  | √ | √ | √ | √ | √ | √ | √ | √ | √ | √ | √ | √ | √ | √ |  |
| HS HK 2154 | √ | √ | √ | √ | √ | √ |  | √ | √ | √ | √ | √ | √ | √ | √ |  |  | √ | √ | √ | √ | √ | √ |  |  |  |  |
| *HPHH HK411* | √ | √ | √ | √ | √ | √ | √ | √ | √ | √ | √ | √ | √ | √ | √ |  | √ | √ | √ | √ | √ | √ | √ |  |  |  |  |
| **HSO 129PT** | √ | √ | √ | √ | √ | √ |  |  |  | √ | √ |  | √ | √ | √ |  | √ | √ |  | √ | √ | √ | √ | √ | √ | √ |  |
| **HSO 2336** | √ | √ | √ | √ | √ | √ |  |  |  | √ | √ |  | √ | √ | √ |  | √ | √ |  | √ | √ | √ | √ | √ | √ | √ |  |

Abbreviations used in the table: bold – denotes completed genomes, HPI = H. parainfluenzae; HD= H. ducreyi, HH= H. haemolyticus; HP= H. parasuis; HPH=H. parahaemolyticus; HA = H. aegyptius; HPT = H. pittmaniae; HS = H. sputorum; HPHH = H. paraphrohaemolyticus; HSO = H. somnus

Gene abbreviations used: pyruvate dehydrogenase complex, *aceF*; Pyruvate formate lyase, *pflA*; Acetate kinase , *ackA*; Lactate dehydrogenase, *ldhA*; Formate dehydrogenase, *fdxG*; NADH dehydrogenase, *ndh*; NADH dehydrogenase, *nqr*; L-Lactate dehydrogenase^+^, *lldD*; D-Lactate dehydrogenase^+^, *dld*; Cytochrome *bd* oxidase, *cydA*, *cydB*; DMSO reductase, *dmsA*; Nitrate reductase, *napA*; TMAO reductase, *torZ*; Nitrite reductase, *nrfA*; Fumarate reductase^‡^, *frdA;*  isocitrate dehydrogenase, *idh*; citrate synthase, *gltA*; aconitase, *acn*; succinate ddehdyrogenase *sdh*.
