# Supplementary material for: Metabolic versatility in Haemophilus influenzae: a metabolomic and genomic analysis
Source: Front Microbiol. 2014 Mar 4;5:69. doi: 10.3389/fmicb.2014.00069 (PMC3941224; doi:10.3389/fmicb.2014.00069)
Supplement: Table S1 — Chemical shift multiplicity and signal regions used for metabolite identification and quantification. [file DataSheet1.ZIP › 75817_Kappler_Suppl_Table_5.DOCX]

**Table S5 *H. influenzae* RD NMR metabolites detected in the growth medium of samples taken in late exponential growth phase. N.d. – not detected.**

| Metabolite | | Initial conc (mM) | Aerobic (mM) | Microaerophilic (mM) | Anaerobic (mM) |
| --- | --- | --- | --- | --- | --- |
| **Substrates** | Glucose | 10 | 8.326 | 4.228 | 4.082 |
|  | Pyruvate | 0.87 | 0.031 | 0.040 | 0.025 |
|  | Inosine | 6.5 | 0.866 | 2.444 | 2.689 |
| **Amino Acids** | Glycine | 0.133 | 4.371 | 2.983 | 1.040 |
|  | Threonine | 0.169 | 0 | 0.032 | 0.023 |
|  | Hydroxyproline | 0.153 | 0.267 | 0.178 | 0.119 |
|  | Proline | 0.174 | 0.208 | 0.171 | 0.230 |
|  | Methionine | 0.101 | 0.049 | 0.061 | 0.039 |
|  | Valine | 0.171 | 0.032 | 0.073 | 0.068 |
|  | Isoleucine | 0.382 | 0.053 | 0.107 | 0.119 |
|  | Leucine | 0.382 | 0.010 | 0.090 | 0.124 |
|  | Phenyalanine | 0.09 | 0.009 | 0.007 | 0.024 |
|  | Tyrosine | 0.111 | 0.006 | 0.013 | 0.027 |
| **Products** | Formate | n.d. | 1.86 | 4.05 | 23.36 |
|  | Acetate | n.d. | 11.61 | 6.43 | 4.28 |
|  | Hypoxanthine | n.d. | 3.53 | 1.75 | 0.701 |
|  | Glycerol | n.d. | 0.856 | 0.583 | 0.185 |
|  | Succinate | n.d. | 0.011 | 0.034 | 0.641 |
|  | Lactate | n.d. | 0.003 | 0.036 | 0.013 |
| **Other medium components** | Choline | 0.021 | 0.025 | 0.017 | 0.057 |
|  | Uracil | 0.78 | 0.184 | 0.503 | 0.375 |
