# Supplementary material for: Metabolic versatility in Haemophilus influenzae: a metabolomic and genomic analysis
Source: Front Microbiol. 2014 Mar 4;5:69. doi: 10.3389/fmicb.2014.00069 (PMC3941224; doi:10.3389/fmicb.2014.00069)
Supplement: Table S1 — Chemical shift multiplicity and signal regions used for metabolite identification and quantification. [file DataSheet1.ZIP › 75817_Kappler_Suppl_Table_6.DOCX]

**Table S6 *H. influenzae* 2019 NMR metabolites detected in the growth medium of samples taken in late exponential growth phase. n.d. – not detected.**

| Metabolite | | Initial conc (mM) | Aerobic (mM) | Microaerophilic (mM) | Anaerobic (mM) |
| --- | --- | --- | --- | --- | --- |
| **Substrates** | Glucose | 10 | 1.98 | 2.26 | 3.864 |
|  | Pyruvate | 0.87 | 0.018 | 0.015 | 0.041 |
|  | Inosine | 6.5 | 2.60 | 3.043 | 3.347 |
| **Amino Acids** | Glycine | 0.133 | 0.202 | 0.331 | 0.295 |
|  | Threonine | 0.169 | 0.108 | 0.153 | 0.736 |
|  | Hydroxyproline | 0.153 | 0.186 | 0.144 | 0.116 |
|  | Proline | 0.174 | 0.163 | 0.113 | 0.128 |
|  | Methionine | 0.101 | 0.040 | 0.057 | 0.096 |
|  | Valine | 0.171 | 0.148 | 0.130 | 0.139 |
|  | Isoleucine | 0.382 | 0.127 | 0.205 | 0.409 |
|  | Leucine | 0.382 | 0.021 | 0.056 | 0.136 |
|  | Phenyalanine | 0.09 | 0.008 | 0.014 | 0.024 |
|  | Tyrosine | 0.111 | 0.006 | 0.007 | 0.029 |
| **Products** | Formate | n.d. | 2.09 | 13.16 | 23.04 |
|  | Acetate | n.d. | 11.17 | 8.06 | 4.74 |
|  | Hypoxanthine | n.d. | 1.63 | 1.53 | 1.242 |
|  | Glycerol | n.d. | 0.046 | 0.04 | 0.032 |
|  | Succinate | n.d. | 0.007 | 0.401 | 1.009 |
|  | Lactate | n.d. | 0.069 | 0.098 | 0.456 |
| **Other medium components** | Choline | 0.021 | 0.047 | 0.102 | 0.079 |
|  | Uracil | 0.78 | 0.323 | 0.401 | 0.466 |
