# Supplementary material for: Metabolic versatility in Haemophilus influenzae: a metabolomic and genomic analysis
Source: Front Microbiol. 2014 Mar 4;5:69. doi: 10.3389/fmicb.2014.00069 (PMC3941224; doi:10.3389/fmicb.2014.00069)
Supplement: Table S1 — Chemical shift multiplicity and signal regions used for metabolite identification and quantification. [file DataSheet1.ZIP › 75817_Kappler_Suppl_Table_1.DOCX]

**Table S1: Chemical shift multiplicity and signal regions used for metabolite identification and quantification**

| **Metabolite** | **^1^H chemical shifts [ppm] multiplicity** | **Chemical region of signal used for concentration determination** |
| --- | --- | --- |
| Glucose | 5.22d; 4.64d; 3.89m; 3.72m; 3.71m; 3.53m; 3.48t; 3.24m | 4.63-4.62 |
| Pyruvate | 2.36s | 2.37-2.35 |
| Inosine | 8.33s; 8.22s; 6.09d; 4.43t; 4.27m | 6.09-6.08 |
| Glycine | 3.56s | 3.57-3.54 |
| Threonine | 1.32s | 1.31-1.30 |
| Hydroxyproline | 4.34m | 4.36-4.34 |
| Proline | 4.12m; 3.41m; 3.33m; 2.34m; 2.06m; 2.02m; 1.97m | 4.14-4.12 |
| Methionine | 2.13s | 2.14-2.12 |
| Valine | 3.6d; 1.03d; 0.98d | 0.99-0.97 |
| Isoleucine | 1.46m; 1.25m; 1.00d; 0.93t | 1.02-0.99 |
| Leucine | 3.72m; 1.74m; 1.69m; 1.66m; 0.96d; 0.93m | 0.96-0.94 |
| Phenyalanine | 7.41m; 7.37m; 7.32t; 3.99m; 3.27m; 3.11m | 7.41-7.39 |
| Tyrosine | 7.19d; 6.88d; 5.11m; 3.19m; 3.04m | 6.90-6.88 |
| Formate | 8.45s | 8.47-8.42 |
| Acetate | 1.91s | 1.93-1.89 |
| Hypoxanthine | 8.20s; 8.19d | 8.19-8.17 |
| Glycerol | 3.78m; 3.65m; 3.55m | 3.65-3.64 |
| Succinate | 2.40s | 2.41-2.39 |
| Lactate | 1.32d | 1.33-1.32 |
| Choline | 4.05m; 3.62m; 3.19s | 3.20-3.18 |
| Uracil | 7.53d | 7.54-7.52 |
